# Neurovisual rehabilitation in multiple sclerosis: Why a close integration of low-vision rehabilitation and neuropsychological rehabilitation may be effective for visual complaints

**DOI:** 10.1177/02692155231210968

**Published:** 2023-11-03

**Authors:** FE van der Feen, GA de Haan, I van der Lijn, DJ Heersema, JF Meilof, J Heutink

**Affiliations:** 1 Clinical and Developmental Neuropsychology, University of Groningen, Groningen, The Netherlands; 2 Royal Dutch Visio, Centre of Expertise for Blind and Partially Sighted People, Huizen, The Netherlands; 3 Department of Neurology, University of Groningen, 10173 University Medical Centre Groningen, Groningen, The Netherlands; 4 MS Centrum Noord Nederland, Groningen, The Netherlands; 5 Department of Neurology, Martini Hospital Groningen, Groningen, The Netherlands

**Keywords:** multiple sclerosis, visual complaints, neurovisual rehabilitation, neuropsychological rehabilitation, low-vision rehabilitation

## Abstract

**Objective:**

The quality of life of people with multiple sclerosis (MS) is often affected by visual complaints. A previous study suggested that visual complaints are not likely to be related to specific visual functions, but by a global decline of cognitive and visual functioning. In this study, we further explore this hypothesis, by investigating the relation between visual functions and global cognitive functioning, aiming to provide recommendations for rehabilitation for visual complaints.

**Design:**

Cross-sectional study.

**Setting:**

A rehabilitation centre for partially sighted and blind people and a MS centre at a university hospital.

**Participants:**

102 people with MS.

**Main measure:**

Correlations between assessments of visual functions (acuity, contrast sensitivity, visual field, smooth pursuit and saccades) and composite scores of a neuropsychological assessment (tests with a visual component and without a visual component).

**Results:**

All composite scores correlated with visual acuity, contrast sensitivity and the sensitivity of the monocular field, but not with smooth pursuit and saccades. Similar patterns were found in various subgroups. Results showed that visual functions that related to visual complaints correlated with a diffuse decline of global cognitive functioning and that visual and cognitive functioning may decline concurrently in people with MS.

**Conclusions:**

Visual complaints may occur as a result of a diffuse decline of the integrity of a cerebral network involved in vision and cognition. People with MS with visual complaints may benefit from neurovisual rehabilitation, in which low-vision rehabilitation and neuropsychological rehabilitation are closely intertwined.

## Introduction

There is an increasing awareness of self-reported visual complaints in people with acquired brain injury. These complaints, including blinded by bright light, needing more time or more light and depth perception difficulties, are highly prevalent in people with multiple sclerosis (MS)^1^ and significantly impact activities and participation in daily life.^2,3^ Rehabilitation focusing on reducing the impact of visual complaints may therefore be highly beneficial. A better understanding of visual complaints can guide healthcare professionals in tailoring rehabilitation. A previous study exploring visual complaints in MS suggested that the extent to which visual complaints may be explained by specifically affected ophthalmological, visual or cognitive functions seems limited.^1,4^ Instead, the results prompted the hypothesis that visual complaints may be indicative of overall reduced cognitive capacity. Reduced contrast sensitivity, visual acuity, sensitivity of the visual field and visual motor speed can indicate a decline in global cognitive functioning.^4,5^ Thus, besides being a direct consequence of visual function impairment, visual complaints might be explained by more diffuse decline or loss of integrity of a complex functional cerebral network. This diffuse decline refers to a global reduction in cognitive or visual functioning, as opposed to distinctive visual or cognitive function disorders. Previous literature regarding the relation between visual and cognitive functioning shows correlations between visual functions and cognitive tests relying on vision as well as cognitive tests not relying on vision in people with MS.^6–10^ This suggests that a decline of visual and cognitive functions may indeed share a common origin.

We further explore the relationship between global cognitive functioning and lower-order visual functions. We focus on visual acuity, contrast sensitivity and the sensitivity of the monocular visual fields, functions relating to visual complaints and global cognitive decline.^4^ We also investigate the relationship between global cognitive decline and smooth pursuit and saccades, visual functions commonly affected in people with MS, but unrelated to visual complaints. We aim to explore the relationship between global cognitive functioning and visual functions, as indicators of loss of integrity of a possibly complex functional cerebral network for vision and cognition. We hypothesize that decline of global cognitive functioning is primarily related to visual functions related to visual complaints. This would provide further evidence that loss of integrity plays an important role in visual complaints. We do not expect smooth pursuit and saccades to be related to global cognitive functioning, since these visual functions are not related to visual complaints and more likely stem from focal lesions. This study may provide further insights in the origin of visual complaints and may support taking next steps towards improving rehabilitation approaches for visual complaints in MS.

## Methods

For this study, we used the data set of a previous study,^4^ to address specific additional hypotheses.

### Participants and procedure

Two groups of people with MS were included in the study. The first group were people with MS who were referred to Royal Dutch Visio (Noord), a centre of expertise for blind and partially sighted people offering visual rehabilitation for people with visual disorders following acquired brain damage. The Screening Visual Complaints questionnaire,^11^ a questionnaire that screens for visual complaints, was used to determine the presence and nature of visual complaints. People who were referred for visual complaints between July 2017 and December 2020 (data collection ended in 2020 because a total cohort of people with MS filled out the Screening Visual Complaints questionnaire and was given the chance for referral to Visio) and had at least one complaint on the Screening Visual Complaints questionnaire often/always, or a discomfort score of ≥6, were included in this study. The second group consisted of people with MS with no or minimal visual complaints (no complaints often/always, ≤5 complaints sometimes and a discomfort score of ≤4) on the Screening Visual Complaints questionnaire. They were included as a control group in our previous study^4^ and were not referred to Royal Dutch Visio. People from the second group were recruited from the MS Centrum Noord Nederland. These two groups were included to ensure a wide and continuous range of the number of visual complaints. All participants received a protocolled visual functions assessment and a neuropsychological assessment; referred people as part of their standard rehabilitation programme, people in the control group only for research purposes. Referred people in whom no visual function assessment or neuropsychological assessment had taken place, because performing the assessment was not considered to be of importance for the rehabilitation, were not included in the study. Demographic and medical data of all participants were retrieved from electronic patient files. All participants gave written consent to participate in the study and use their data. Ethical approval was provided by the Medical Ethics Committee of the University Medical Centre Groningen (NL62728.042.17).

### Materials

Visual acuity was assessed with the EDTRS letter chart,^12^ at 4 m with 500 lux and LogMAR scores were used for the analyses. Either the Vistech^13^ or the Gecko^14^ test was used for contrast sensitivity, using the LogCS scores in the analyses. Sensitivity of the monocular visual fields was assessed with the Humphrey Field Analyzer (HFA), using the 24-2 SITA fast protocol.^15^ Smooth pursuit and saccades were assessed by a trained orthoptist. For smooth pursuit, participants were asked to follow a light, moving horizontally or vertically. To assess saccades, the participants were asked to look alternatively at one of two objects that were 40 cm apart. For the sensitivity of the visual field, a score of 2 (both eyes impaired), 1 (one of the eyes impaired) or 0 (none of the eyes impaired) was attributed based on the HFA score (impaired = dB < −3.00). For both smooth pursuit and saccades, also a score of 0, 1 or 2 was attributed (0 = not impaired, 1 = impaired horizontally or vertically, 2 = impaired both horizontally and vertically).

The DiaNAH-battery^16^ was administered for assessing visual perception. This battery is specifically designed to assess visual perception in people with acquired brain injury. The Bells Test, Taylor Complex Figure, TMT A and B, Dot Counting Task, Crowding Task, Corsi Blocks (span) and Silhouettes were used for this study. All tests were administered digitally. We also included auditory-based cognitive tests, which we will refer to as non-visual cognition tests in this study. The non-visual cognitive tests consisted of the DAT Letter Fluency^17^ (total score), the Digit Span (forward, backward and sorting)^18^ and 15 Words Test (retention and delayed recall).^19^ See Table 1 for the tests and test scores that were used.

**Table 1.** Tests and used tests scores that were included in the composite scores.

Composite score	Test	Used raw scores
Visual perception^a^	Bells Test	Total no. of correctly crossed bells
	Taylor Complex Figure	Total no. of correctly placed elements
	Dot Counting Task	Total no. of correctly counted trials
	Crowding Task	Total no. of correctly named letters
	Corsi Blocks	Max span of correctly repeated series
	Silhouettes	Total no. of correctly recognized shapes
Non-visual cognition^a^	DAT Letter Fluency	Total no. of correctly named words
	Digit span	Total no. of correctly repeated series
	15 Words Test	Total no. of correctly retained words
		Total no. of correctly recalled words
TMT	TMT A	Time to complete TMT A
	TMT B	Time to complete TMT B

No: number; TMT: trail-making test.

a The combined visual perception and non-visual cognition composite score was calculated by summing the visual perception and non-visual cognition composite score.

### Data analyses

Because it was not always possible to complete all tests of the visual functions or neuropsychological assessment, there were missing values. For example due to time constraints, covid-19 constraints, or when the participant experienced the tests as too strenuous. For people in certain wheelchairs, the HFA could not be performed. The composite scores of the neuropsychological assessment could only be calculated for people who completed all tests needed for the different composite scores. The sample sizes therefore varied among the different correlations that were calculated.

All analyses were performed in IBM SPSS v26.0. To operationalize global cognitive functioning, four composite scores were calculated (see Table 1). First a ‘visual perception composite score’ and a ‘non-visual cognition composite score’ were calculated. We calculated these separately to be able to distinguish between tests aimed at assessing visual perception and tests that aimed to assess cognitive functions with no visual component. To do so, all test scores were transformed to a scale from 0 to 100. The transformed scores were summed to attain the composite scores. The visual perception and non-visual cognition composite scores were summed for a combined visual perception and non-visual cognition composite score (‘combined composite score’). Since our previous study showed motor speed to be an important indicator of visual complaints,^4^ and the TMT comprises multiple visual and non-visual cognitive components, we calculated a TMT composite score separately, by summing the scores on the TMT A and TMT B. To prevent overestimation of the correlation coefficient, participants (*n* = 4) with outlier TMT sum scores (>3 SD) were identified and were not included in the calculation of the TMT composite score. There were no outlier scores in the visual perception or non-visual cognition tests.

Bootstrapped partial Pearson's correlations (1000 samples; small: 0.10–0.29, moderate: 0.30–0.49, large:≥0.5^20^) were calculated between the composite scores and visual acuity, contrast sensitivity, visual field, smooth pursuit and saccades. The bootstrapped ranges of scores were also calculated. In partial correlations, we controlled for age and sex because these variables could influence both cognition and lower-order visual functions.

We also divided the total groups into subgroups which were: (1) people with MS with visual complaints and with no or minimal visual complaints (see *Participants and Procedure*); (2) people with MS with a history of optic neuritis and people with MS without a history of optic neuritis; (3) people with relapsing-remitting MS and people with secondary progressive MS and (4) people with a low (lowest 25% in our sample) EDSS score^21^ and people with a higher EDSS (highest 25% is our sample). The relationship between visual functions and cognitive functioning was compared within these four different subgroups, for further insight into this relationship with other MS-related variables. The partial correlations (controlled for age and sex) were calculated in each subgroup.

A Fisher's Z-transformation^22^ was performed on all correlation coefficients, transforming the correlations into z-scores (formula: z′ = 0.5[ln(1 + r) − ln(1 − r)]). Using the z-scores, the Z-observed (Z-obs) was calculated per comparison, using the formula Z-obs = (z1 − z2)/(sqrt(1/(n1 − 3) + 1/(n2 − 3)). If the outcome is smaller than −1.96 or larger than 1.96, this means that the two correlations are significantly different from each other (α < 0.05).

## Results

### Participants

Seventy-one people with MS followed a rehabilitation programme at Royal Dutch Visio. Six people were excluded from the analyses because either no visual functions assessment or neuropsychological assessment was performed during the rehabilitation programme. All 37 people who were included in the control group (people with no or minimal visual complaints) participated in both assessments. In total, 102 people with MS were included in the analyses, of whom the demographic and clinical characteristics are shown in Table 2.

**Table 2.** Demographical and clinical characteristics.

People with MS (*n* = 102)
Age (*M*, *SD*)	52.3 (12.2)
Female (*n*, %)	75 (73.5)
Education (*n*, %)^a^, Low/intermediate/high	7 (6.9)/ 49 (48.0)/ 46 (45.1)
Type of MS (*n*, %), RRMS/SPMS/PPMS; Other/unknown	50 (49.0)/ 34 (33.3)/ 12 (11.8); 3 (2.9)/ 3 (2.9
Disease duration (*M*, *SD*); Unknown (n, %)	13.69 (10.0); 3 (2.9)
EDSS^b^ (21) (*n*, %); Mild/Moderate/Severe/Very severe; Unknown	36 (35.3)/ 29 (28.4)/ 19 (18.6)/ 0 (0); 18 (17.6)
History of ON (*n*, %); Unknown	27 (26.5); 14 (13.7)
Visual Acuity (min − max) (EDTRS(12))	0.32–2.0

EDSS: Expanded Disability Status Score; EDTRS: early treatment of diabetic retinopathy study; M: mean; MS: multiple sclerosis; ON: optic neuritis; PPMS: primary progressive multiple sclerosis; RRMS: relapsing-remitting multiple sclerosis; SD: standard deviation; SPMS: secondary progressive multiple sclerosis.

^a^ Education level was based on the classification by de Vent et al. (2018). Low: 1–4, intermediate: 5, high: 6–7.

^b^ EDSS categories: mild: 0–3.0, moderate: 3.5–5.0, severe 5.5–7, very severe: 7+.

### Correlations and group comparisons

**Figure 1.**
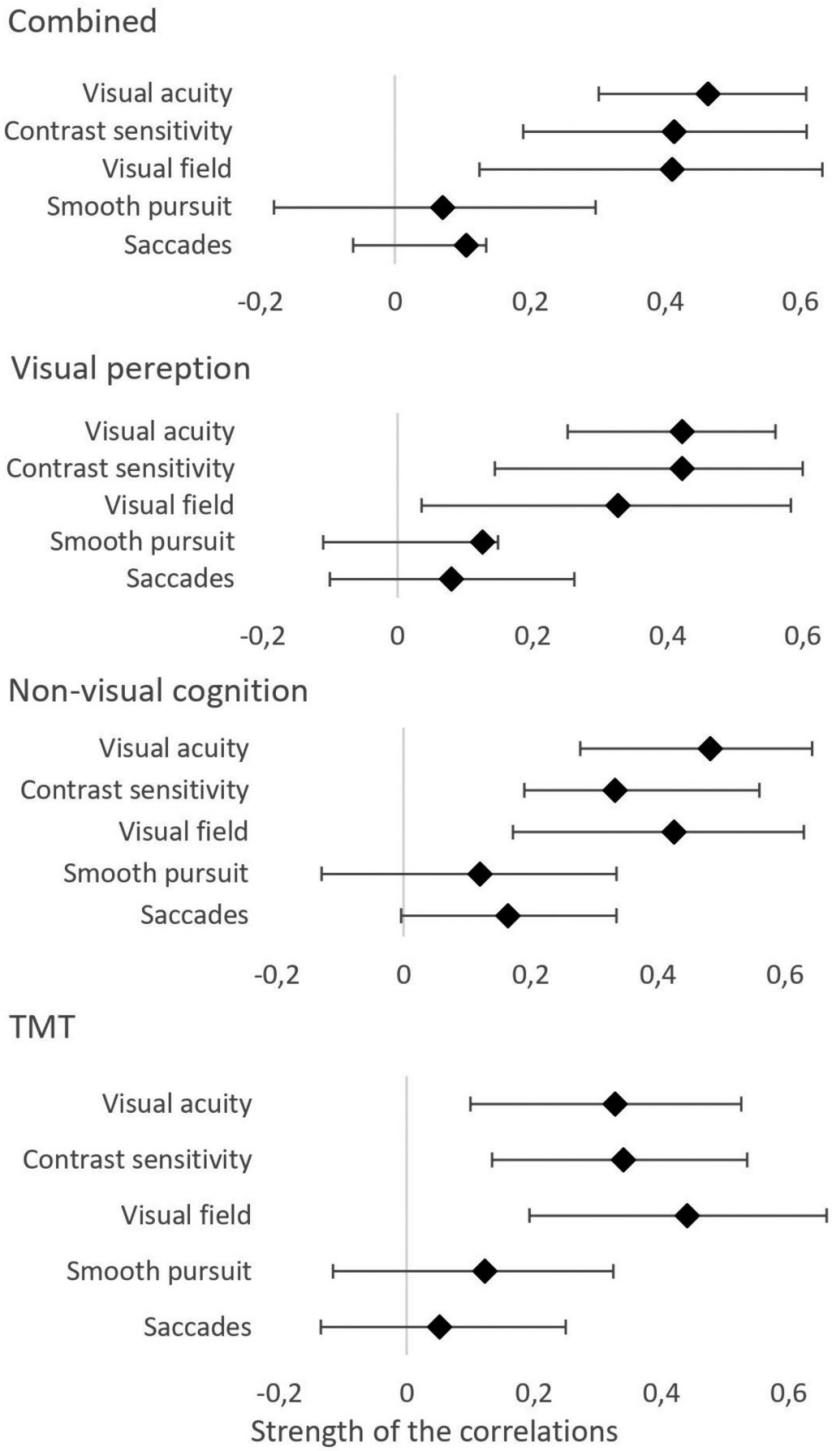
Values and ranges of bootstrapped correlations.

The correlations for the full group are shown in Table 3 and Figure 1. For all correlations found, a greater degree of visual function decline meant a greater degree of global cognitive decline. Visual acuity, contrast sensitivity and visual field all showed moderate and significant correlations with the total composite score of the neuropsychological assessment, composite visual perception score, the composite cognition score and the composite TMT score. The TMT score showed a significant, but small correlation with smooth pursuit. The other scores of the neuropsychological assessment were not related to smooth pursuit or saccades.

**Table 3.** Bootstrapped partial correlations between cognitive composite scores and visual functions.

	Combined composite	Visual perception composite	Non-visual cognition composite	TMT composite
Visual acuity	.464***	.422***	.483***	.328***
Contrast sensitivity	.414***	.422***	.333***	.341***
Visual field	.411**	.327*	.426**	.441**
Smooth pursuit	.071	.127	.120	.123*
Saccades	.106	.080	.164	.052

^a^ Controlling for age and sex.

*p < .05 **p < 0.01, ***p < 0.001.

For the correlations and comparisons of the correlations in the four subgroups, see the supplementary materials (Supplemental Table 1). Overall, the correlations within these subgroups were consistent with the correlations in the total group. No differences were found between the referred group and the control group or between the optic neuritis and no-optic neuritis groups. No comparison could be made between the referred group and control group regarding correlations with visual field, because no impaired visual fields were found in the control group. Regarding the comparisons between people with relapsing-remitting MS and secondary progressive MS and people with a low EDSS and high EDSS, no statistical differences were found either. However, except for correlation regarding ON, the correlations were stronger in people with visual complaints, people with secondary progressive MS and people with a high EDSS.

## Discussion

The purpose of the present study was to further understand the role of a complex cerebral network possibly explaining visual complaints and to gather additional insights for determining the best rehabilitation to minimize the impact of the complaints. Results showed a relationship between visual complaints and global cognition. The correlations were not restricted to cognitive measures relying on vision. Visual functions also correlated with non-visual cognitive functioning.

In line with our expectations, the results of the correlation analyses show that global cognitive functioning was related to visual acuity, contrast sensitivity and sensitivity of the monocular visual field, but not to smooth pursuit and saccades. This shows that not merely tests that aim to assess visual perception relate to visual functioning. This demonstrated a diffuse and concurrent decline of cognitive functioning and afferent visual functions. Clinically meaningful and observable decline of smooth pursuit and saccades were not related to global cognition in our study. This may be because disorders of smooth pursuit and saccades primarily originate from focal brain stem damage, not affecting the cerebral network.^23^ However, other studies suggested subtle, subclinical deviations of eye movements were associated with focal and global grey matter atrophy.^24^

Regarding the analyses within the subgroups, we need to point out that the sample sizes were small. The bootstrapping of the analyses showed that the power of these analyses was not sufficient and the correlations may be unreliable, so we need to interpret these results with caution. Correlations between visual functioning and global cognitive functioning were stronger in people with visual complaints, people with secondary progressive MS and in people with higher EDSS, indicating that with further disease progression, visual functioning and global cognitive functioning seem to decline concurrently. Regarding the correlations of smooth pursuit and saccades with global cognitive functioning, no significant correlations were found in any of the groups. The results of the study strengthen the hypothesis that visual complaints in people with MS may be a manifestation of a diffuse decline or the loss of integrity of the cerebral network involved in vision and cognition.

There may be alternative explanations and limitations. It is unlikely that the results are fully explained by declined visual functioning negatively affecting the cognitive testing. However, cognitive functioning may have negatively influenced visual field testing,^25^ since reliably performed HFA requires substantial cognitive effort, especially regarding sustained attention, motor speed and reaction time. These cognitive domains can be affected in people with MS.^26^ In our study, 40–42% of the participants showed an increased reaction time and 54–68% an increased motor speed (we did not assess sustained attention). However, all HFA measurements classified as unreliable were excluded from the analysis. Inevitably, this caused some missing values that are not likely to be completely at random. We excluded the participants only from the correlation analysis regarding visual field, but not from other analyses, to maximize sample size and minimize bias. Still, although bootstrapping the analyses showed the correlations to be reliable, the bootstrapped range of scores around the correlation were rather wide. The results should be replicated in a larger sample in order to assure external reliability.

The results of this study and previous literature show that cognitive functioning and visual functioning may be integrated and that a diffuse decline of this possibly integrated network may be related to visual complaints, as opposed to distinctive or isolated visual and cognitive disorders. These insights have some implications for the treatment of people with MS reporting visual complaints. Currently, compensation and adaptation strategies are typically applied since improvement or restoration of an impaired function is often unattainable.^27,28^ Compensatory strategies, aimed at learning an individual to do a task or activity as well as possible despite loss of a particular function by using (relatively) intact functions, are usually based upon a strengths and weaknesses assessment regarding specific visual functions or cognitive domains.^29,30^ This approach may not be beneficial for people with MS, when complaints are not clearly a result of specific functional loss, but of a general decline or loss of integrity of a complex network involving visual and cognitive processes. Compensation or adaptation strategies could improve vision in daily life, but may not be sufficient in reducing visual complaints. Instead, a general functional decline may call for more general approaches. Regarding rehabilitation for people with MS with visual complaints that are not caused by specific functional disorders, we propose ‘neurovisual rehabilitation’. Neurovisual rehabilitation focuses on vision problems that are a result of changes in the brain, due to acquired or traumatic brain injury or normal ageing. Moreover, low-vision rehabilitation (aimed at reducing the impact of visual function or ophthalmological disorders) and neuropsychological rehabilitation (for people with affected cognitive functions) are closely integrated. Rehabilitation could mainly focus on reducing the burden on the integrated networks. Adjustment or accommodation of the environment is often only applied when other strategies turn out to be insufficient. However, just this could be a key intervention in the rehabilitation for people with MS with visual complaints. For example, applying contrast and logically structuring surroundings will make the visual world more easily visible, and thus less selective or sustained attention is required to see and less (cognitive) effort is needed to perceive or act on stimuli. Such affect has been shown previously in people with Alzheimer's disease, where enhancing the visual stimulus improved cognitive functioning.^31^

In clinical care, one could argue that when diffuse decline plays an important role, searching for specific function disorders may not be meaningful in understanding visual complaints. Extensive testing may furthermore place an unnecessary burden on people with MS. Hence, we would suggest to only shortly screen for function disorders. The TMT could provide a quick indication of global functioning.^32^ A short visual and cognitive assessment could rule out ophthalmological, visual or cognitive disorders and can assure optimal refraction, to optimize incoming input from the visual world. If the rehabilitation programme does not yield effective reduction of the visual complaints, further assessments can be done to support rehabilitation.

**Clinical messages**

- Visual and cognitive functioning show a concurrent, diffuse decline in people with MS.
- People with MS with visual complaints may benefit from neurovisual rehabilitation, in which low-vision rehabilitation and neuropsychological rehabilitation are closely intertwined.
- Neurovisual strategies for visual complaints should be aimed at reducing the burden on the integrated vision and cognition networks.

## Supplemental Material

sj-docx-1-cre-10.1177_02692155231210968 - Supplemental material for Neurovisual rehabilitation in multiple sclerosis: Why a close integration of low-vision rehabilitation and neuropsychological rehabilitation may be effective for visual complaints Click here for additional data file. Supplemental material, sj-docx-1-cre-10.1177_02692155231210968 for Neurovisual rehabilitation in multiple sclerosis: Why a close integration of low-vision rehabilitation and neuropsychological rehabilitation may be effective for visual complaints by FE van der Feen, GA de Haan, I van der Lijn, DJ Heersema, JF Meilof and J Heutink in Clinical Rehabilitation
