# Supplementary material for: Neurovisual rehabilitation in multiple sclerosis: Why a close integration of low-vision rehabilitation and neuropsychological rehabilitation may be effective for visual complaints
Source: Clin Rehabil. 2023 Nov 3;38(3):393–402. doi: 10.1177/02692155231210968 (PMC10829419; doi:10.1177/02692155231210968)
Supplement: sj-docx-1-cre-10.1177_02692155231210968 - Supplemental material for Neurovisual rehabilitation in multiple sclerosis: Why a close integration of low-vision rehabilitation and neuropsychological rehabilitation may be effective for visual complaints [file sj-docx-1-cre-10.1177_02692155231210968.docx]

**Supplementary Materials**

**Table 1: Correlations between visual acuity and cognitive composite scores of the referred group and control group, people with RRMS and SPMS, people with a history of ON and without a history of ON, and people with low EDSS and people with high EDSS, including comparison tests**

| **Group** | **Referred** | | *N* | | **Control** | | *N* | *Z*-observed | *p* |
| --- | --- | --- | --- | --- | --- | --- | --- | --- | --- |
| **Acuity** |  | |  | |  | |  |  |  |
| Combined | 0.430 | | 57 | | 0.400 | | 35 | 0.162 | 0.871 |
| Visual perception | 0.407 | | 59 | | 0,227 | | 35 | 0,907 | 0,364 |
| Non-visual cognition | 0.511 | | 62 | | 0.321 | | 37 | 1.074 | 0.283 |
| TMT | 0.449 | | 62 | | 0.260 | | 36 | 1.000 | 0.317 |
| **Contrast** |  | |  | |  | |  |  |  |
| Combined | 0.377 | | 54 | | 0.312 | | 35 | 0.327 | 0.743 |
| Visual perception | 0.324 | | 55 | | 0.33 | | 35 | -0.030 | 0.976 |
| Non-visual cognition | 0.446 | | 59 | | 0.101 | | 37 | 1.740 | 0.082 |
| TMT | 0.356 | | 58 | | 0.279 | | 36 | 0.389 | 0.697 |
| **Visual field** |  | |  | |  | |  |  |  |
| Combined | 0.375 | | 40 | | - | | - | - | - |
| Visual perception | 0.263 | | 41 | | - | | - | - | - |
| Non-visual cognition | 0.459 | | 41 | | - | | - | - | - |
| TMT | 0.346 | | 42 | | - | | - | - | - |
| **Smooth pursuit** |  | |  | |  | |  |  |  |
| Combined | 0.112 | | 56 | | 0.115 | | 35 | -0.014 | 0.989 |
| Visual perception | 0.131 | | 58 | | 0.109 | | 35 | 0.100 | 0.920 |
| Non-visual cognition | 0.182 | | 60 | | 0.098 | | 37 | 0.396 | 0.692 |
| TMT | 0.243 | | 61 | | 0.012 | | 36 | 1.082 | 0.279 |
| **Saccades** |  | |  | |  | |  |  |  |
| Combined | 0.113 | | 58 | | 0.154 | | 35 | -0.188 | 0.851 |
| Visual perception | 0.008 | | 60 | | 0.254 | | 35 | -1.139 | 0.255 |
| Non-visual cognition | 0.237 | | 62 | | 0.088 | | 37 | 0.712 | 0.476 |
| TMT | 0.164 | | 62 | | 0.116 | | 36 | 0.225 | 0.822 |
| **Group** | **RRMS** | *N* | | **SPMS** | | | *N* | *Z*-observed | *p* |
| **Acuity** |  |  | |  | | |  |  |  |
| Combined | 0.228 | 46 | | 0.47 | | | 28 | -1.105 | 0.269 |
| Visual perception | 0.190 | 47 | | 0.527 | | | 29 | -1.591 | 0.112 |
| Non-visual cognition | 0.312 | 48 | | 0.509 | | | 31 | -0.991 | 0.322 |
| TMT | 0.244 | 48 | | 0.59 | | | 32 | -1.800 | 0.072 |
| **Contrast** |  |  | |  | | |  |  |  |
| Combined | 0.311 | 44 | | 0.382 | | | 28 | -0.318 | 0.750 |
| Visual perception | 0.304 | 45 | | 0.428 | | | 28 | -0.568 | 0.570 |
| Non-visual cognition | 0.260 | 46 | | 0.34 | | | 32 | -0.366 | 0.714 |
| TMT | 0.190 | 46 | | 0.39 | | | 30 | -0.894 | 0.371 |
| **Visual field** |  |  | |  | | |  |  |  |
| Combined | 0.242 | 35 | | 0.382 | | | 28 | -0.583 | 0.560 |
| Visual perception | 0.095 | 36 | | 0.428 | | | 28 | -1.366 | 0.172 |
| Non-visual cognition | 0.259 | 35 | | 0.34 | | | 32 | -0.347 | 0.728 |
| TMT | 0.202 | 36 | | 0.39 | | | 30 | -0.798 | 0.425 |
| **Smooth pursuit** |  |  | |  | | |  |  |  |
| Combined | 0.001 | 47 | | 0.06 | | | 28 | -0.236 | 0.814 |
| Visual perception | 0.094 | 48 | | 0.095 | | | 29 | -0.004 | 0.997 |
| Non-visual cognition | 0.064 | 48 | | 0.191 | | | 32 | -0.543 | 0.587 |
| TMT | 0.108 | 49 | | 0.211 | | | 31 | -0.441 | 0.659 |
| **Saccades** |  |  | |  | | |  |  |  |
| Combined | 0.056 | 47 | | 0.178 | | | 29 | -0.501 | 0.617 |
| Visual perception | 0.084 | 48 | | 0.148 | | | 30 | -0.267 | 0.790 |
| Non-visual cognition | 0.219 | 49 | | 0.057 | | | 32 | 0.698 | 0.485 |
| TMT | 0.034 | 49 | | 0.059 | | | 31 | -0.105 | 0.917 |
| **Group** | **NON** | *N* | | **NO** | | | *N* | *Z*-observed | *p* |
| **Acuity** |  |  | |  | | |  |  |  |
| Combined | 0.306 | 55 | | 0.428 | | | 26 | -0.564 | 0.573 |
| Visual perception | 0.328 | 55 | | 0.383 | | | 27 | -0.255 | 0.799 |
| Non-visual cognition | 0.426 | 60 | | 0.423 | | | 26 | 0.015 | 0.988 |
| TMT | 0.64 | 59 | | 0.017 | | | 26 | 2.993 | 0.003* |
| **Contrast** |  |  | |  | | |  |  |  |
| Combined | 0.3 | 52 | | 0.432 | | | 26 | -0.605 | 0.545 |
| Visual perception | 0.319 | 52 | | 0.375 | | | 27 | -0.256 | 0.798 |
| Non-visual cognition | 0.36 | 57 | | 0.432 | | | 26 | -0.343 | 0.731 |
| TMT | 0.551 | 56 | | 0.469 | | | 26 | 0.445 | 0.657 |
| **Visual field** |  |  | |  | | |  |  |  |
| Combined | 0.242 | 40 | | 0.499 | | | 16 | -0.934 | 0.350 |
| Visual perception | 0.157 | 40 | | 0.542 | | | 17 | -1.430 | 0.153 |
| Non-visual cognition | 0.369 | 42 | | 0.436 | | | 16 | -0.250 | 0.803 |
| TMT | 0.438 | 42 | | 0.362 | | | 17 | 0.291 | 0.771 |
| **Smooth pursuit** |  |  | |  | | |  |  |  |
| Combined | 0.078 | 54 | | 0.08 | | | 26 | -0.008 | 0.994 |
| Visual perception | 0.065 | 54 | | 0.083 | | | 27 | -0.073 | 0.942 |
| Non-visual cognition | 0.128 | 59 | | 0.07 | | | 26 | 0.237 | 0.813 |
| TMT | 0.36 | 58 | | 0.332 | | | 26 | 0.128 | 0.898 |
| **Saccades** |  |  | |  | | |  |  |  |
| Combined | 0.091 | 56 | | 0.09 | | | 26 | 0.004 | 0.997 |
| Visual perception | 0.062 | 56 | | 0.014 | | | 27 | 0.195 | 0.845 |
| Non-visual cognition | 0.142 | 60 | | 0.168 | | | 26 | -0.108 | 0.914 |
| TMT | 0.078 | 59 | | 0.047 | | | 26 | 0.126 | 0.900 |
| **Group** | **Low EDSS** | *N* | | **High EDSS** | | *N* | | *Z*-observed | *p* |
| **Acuity** |  |  | |  | |  | |  |  |
| Combined | 0.206 | 26 | | 0.618 | | 30 | | -1.807 | 0.071 |
| Visual perception | 0.161 | 26 | | 0.543 | | 32 | | -1.597 | 0.110 |
| Non-visual cognition | 0.217 | 27 | | 0.641 | | 36 | | -2.011 | 0.044* |
| TMT | 0.111 | 48 | | 0.523 | | 35 | | -2.028 | 0.043 |
| **Contrast** |  |  | |  | |  | |  |  |
| Combined | 0.202 | 26 | | 0.363 | | 28 | | -0.607 | 0.544 |
| Visual perception | 0.247 | 26 | | 0.337 | | 29 | | -0.344 | 0.731 |
| Non-visual cognition | 0.086 | 27 | | 0.448 | | 34 | | -1.456 | 0.145 |
| TMT | 0.198 | 27 | | 0.51 | | 32 | | -1.312 | 0.189 |
| **Visual field** |  |  | |  | |  | |  |  |
| Combined | 0.028 | 19 | | 0.436 | | 17 | | -1.200 | 0.230 |
| Visual perception | 0.137 | 19 | | 0.422 | | 18 | | -0.869 | 0.385 |
| Non-visual cognition | 0.061 | 20 | | 0.48 | | 18 | | -1.304 | 0.192 |
| TMT | 0.246 | 20 | | 0.444 | | 19 | | -0.649 | 0.516 |
| **Smooth pursuit** |  |  | |  | |  | |  |  |
| Combined | 0.134 | 27 | | 0.106 | | 29 | | 0.100 | 0.920 |
| Visual perception | 0.012 | 27 | | 0.017 | | 31 | | -0.018 | 0.986 |
| Non-visual cognition | 0.157 | 28 | | 0.036 | | 34 | | 0.455 | 0.649 |
| TMT | 0.247 | 28 | | 0.202 | | 34 | | 0.176 | 0.860 |
| **Saccades** |  |  | |  | |  | |  |  |
| Combined | 0.068 | 27 | | 0.011 | | 30 | | 0.204 | 0.839 |
| Visual perception | 0.019 | 27 | | 0.026 | | 32 | | -0.025 | 0.980 |
| Non-visual cognition | 0.111 | 28 | | 0.091 | | 35 | | 0.076 | 0.940 |
| TMT | 0.056 | 24 | | 0.202 | | 34 | | -0.526 | 0.599 |

EDSS: Expanded Disability Status Score; NON: no optic neuritis; ON: optic neuritis; RRMS: relapsing remitting multiple sclerosis; SPMS: secondary progressive multiple sclerosis; TMT: Trail Making Test
